# Comparison of clinical outcomes of pulmonary nocardiosis between AIDS and non-AIDS patients

**DOI:** 10.1186/s12879-024-09519-2

**Published:** 2024-06-28

**Authors:** Wilawan Thipmontree, Yupin Suputtamonkol

**Affiliations:** 1 Department of Medicine, Maharat Nakhon Ratchasima Hospital, Nakhon Ratchasima, 30000 Thailand; 2 Department of Medicine, Faculty of Medicine Siriraj Hospital, Mahidol University, Bangkok, 10700 Thailand

**Keywords:** Nocardia, Nocardiosis, AIDS, Pneumonia

## Abstract

**Background:**

Nocardia species can affect both immunocompetent and immunocompromised people.

**Method:**

This retrospective study, from 2009 to 2022, aims to compare the survival analyses of pulmonary nocardiosis in AIDS and non-AIDS patients in northeastern Thailand.

**Results:**

A total of 215 culture-confirmed cases of pulmonary nocardiosis: 97 with AIDS and 118 without AIDS. The median CD4 count of AIDS patients was 11 cells/µL (range: 1–198), and 33% had concurrent opportunistic infections. 63.6% of 118 non-AIDS patients received immunosuppressive medications, 28.8% had comorbidities, and 7.6% had no coexisting conditions. Disseminated nocardiosis and pleural effusion were more prevalent among AIDS patients, whereas non-AIDS patients revealed more shock and respiratory failure. One hundred-fifty patients underwent brain imaging; 15 (10%) had brain abscesses. Patients with pulmonary nocardiosis have overall 30-day and 1-year mortality rates of 38.5% (95% CI: 32.3%, 45.4%) and 52.1% (95% CI: 45.6%, 58.9%), respectively. The Cox survival analysis showed that AIDS patients with disseminated nocardiosis had a 7.93-fold (95% CI: 2.61–24.02, *p* < 0.001) increased risk of death within 30 days compared to non-AIDS patients when considering variables such as age, Charlson comorbidity index, concurrent opportunistic infections, duration of illness, shock, respiratory failure, multi-lobar pneumonia, lung abscesses, and combination antibiotic therapy. While AIDS and pulmonary nocardiosis had a tendency to die within 30 days (2.09 (95% CI, 0.74–5.87, *p* = 0.162)).

**Conclusion:**

AIDS with pulmonary nocardiosis, particularly disseminated disease, is a serious opportunistic infection. Early diagnosis and empiric treatment with a multidrug regimen may be the most appropriate approach in a resource-limited setting.

## Introduction

Nocardiosis is caused by *Nocardia* species, aerobic gram-positive, beaded, branching filament bacteria, which can be found in the environment worldwide [1]. Nocardia species can infect both immunocompetent and immunocompromised people [1, 2]. However, those with human immunodeficiency virus/acquired immunodeficiency syndrome (HIV/AIDS), autoimmune disorders, immunosuppressive medication use, or compromised immune systems are more susceptible to developing the disease [3–5]. Nocardiosis can present in various clinical forms, such as pulmonary, extrapulmonary, and disseminated nocardiosis. The most common manifestation is nocardiosis of the lungs. Isolated *Nocardia* species obtained from respiratory secretions, blood, pus, or tissue specimens by culture or molecular techniques serve to confirm the diagnosis [6, 7]. Trimethoprim-sulfamethoxazole (TMP-SMX) is the mainstay of treatment. Combination therapy is recommended for disseminated nocardiosis, although there are no standard regimens [2]. The clinical outcomes of nocardiosis can vary depending on the site of infection, individual immune status, and antimicrobial therapy.

## Materials and methods

### Patients

A retrospective study of adult patients (≥ 15 years) infected with *Nocardia* species was performed by reviewing patient charts in a 1300-bed tertiary care hospital in northeastern Thailand from January 2009 to December 2022. Patients who had a positive culture showing *Nocardia* species infection at a minimum of one site (e.g., sputum, blood, pus, or body fluid) were included in the study. Statistical analysis excluded patients with extrapulmonary nocardiosis (neck abscess (10), cervical lymphadenopathy (1), mycetoma (1), and corneal ulcer (1)), as well as nine patients who denied the follow-up period.

Disseminated nocardiosis was defined as the presence of *Nocardia* species in the blood culture and/or the presence of at least two non-contiguous organs. Based on their HIV status, we divided the patients into two groups: HIV-positive (97) and HIV-negative (118). We classified all 97 individuals with HIV as having AIDS because their CD4 count was below 200 cells/µL (range 1-198).

The purpose of this study is to compare the survival analysis of pulmonary nocardiosis in patients with and without AIDS.

### Statistical analysis

Descriptive statistics with means and percentages were used, and data were compared using Fisher’s exact test for categorical variables and the Student’s t test for data on normally distributed continuous variables, using two-tailed *P* < 0.05. We used Cox proportional hazards regression models to find risk ratios (HRs) and 95% confidence intervals (CIs) to investigate the correlation between HIV status and 30-day mortality. Confounding variables were accounted for in these models.

## Results

**Fig. 1:**
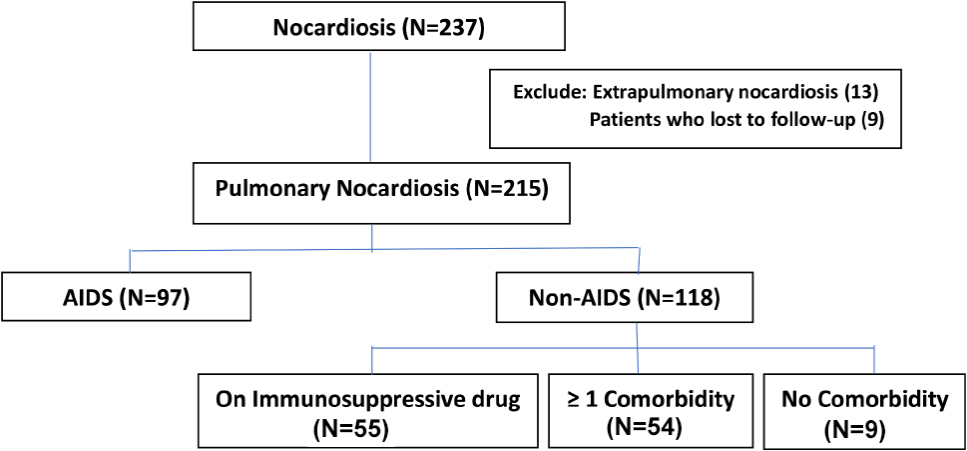
From 2009–2022, 237 culture-confirmed *Nocardia species* infection patients

A total of 237 patients were culture-confirmed with *Nocardia* species infection during the period from 2009 to 2022 (Fig. 1). The study excluded 13 patients who presented with extrapulmonary nocardiosis, including those with neck abscess (10), cervical lymphadenopathy (1), mycetoma (1), and corneal ulcer (1). Additionally, nine patients with pulmonary nocardiosis were excluded due to non-compliance with the follow-up. The final analysis comprised 215 pulmonary nocardiosis cases: 97 with AIDS and 118 without AIDS. Most AIDS patients (94, 97%) had CD4 less than 100 cells/µL. Thirty-two cases (33%) had concurrent opportunistic infections: cryptococcal infection (21), tuberculosis (7), non-tuberculous mycobacterium (2), salmonellosis (1), and esophageal candidiasis (1). Of the 118 non-AIDS patients, 75 cases (63.6%) were treated with immunosuppressive medication, 34 cases (28.8%) had comorbidities, and 9 cases (7.6%) had no coexisting conditions (Table 1).

**Table 1:** Comorbidities of patients with pulmonary nocardiosis

Underlying conditions	*N* = 215 (%)
1. AIDS patients (Median CD4 = 11 cell/µL) (range 1-198)	97 (45.1)
2. Non-AIDS patients	118 (54.9)
2.1 Received Immunosuppressive therapy	
SLE (active disease; LN, AIHA, ITP)	20 (16.9)
COPD, Asthma	20 (16.9)
Hematologic diseases (AIHA, ITP, lymphoma, MM, MDS)	18 (15.5)
Nephrotic syndrome	6 (5.1)
Neurological disease (CIDP, polyneuritis, Tolosa-Hunt syndrome)	6 (5.1)
Connective tissue diseases (RA, Sclerosis, overlapping disease)	4 (3.4)
Post COVID-19 infection (received steroid)	1 (0.8)
2.2 Comorbidities	
Bronchiectasis	11 (9.3)
Chronic liver disease (Alcoholic cirrhosis, CHB, CHC)	9 (7.6)
Diabetic mellitus	7 (5.9)
Heart disease	3 (2.5)
Solid tumor (no chemotherapy for > 1 year)	2(1.7)
Chronic kidney disease	2(1.7)
2.3 No comorbidities	9 (7.6)

Comparing the clinical manifestations of pulmonary nocardiosis in patients with and without AIDS is demonstrated in Table 2. The majority of them were male, with a mean age (± SD) of 37.4 ± 10.19 and 60.9 ± 16.55 years old in AIDS and non-AIDS patients, respectively.

**Table 2:** Clinical manifestations of pulmonary nocardiosis in AIDS and non-AIDS patients

Factors	Total *N* = 215 (%)	AIDS *N* = 97 (%)	Non-AIDS *N* = 118 (%)	*P* value
Male	124 (57.7)	65 (67.0)	59 (50.0)	0.013
Age ≥ 60 years	75 (34.9)	2 (2.1)	73 (61.9)	< 0.001
Charlson comorbidity index, median (IQR)	2 (1,6)	6 (6,6)	1 (0,1)	< 0.001
Charlson comorbidity index ≥ 2	112 (52.1)	97 (100)	15 (12.7)	< 0.001
Duration of illness ≥ 14 days	111 (51.6)	76 (78.4)	35 (29.7)	< 0.001
Shock (on admission)	51 (23.7)	16 (16.5)	35 (29.7)	0.025
Respiratory failure (on admission)	121 (56.3)	40 (41.2)	81 (68.6)	< 0.001
Multi-lobar pneumonia (≥ 2 lobes)	139 (64.6)	53 (54.6)	86 (72.9)	0.006
Lung abscess	52 (24.2)	27 (27.8)	25 (21.2)	0.267
Brain abscess (*N* = 150)	15 (10.0)	5 (6.5)	10 (13.7)	0.178
**Site of infection**				
1. Disseminated nocardiosis	39 (18.1)	21 (21.6)	18 (15.3)	0.286
Brain abscess	15 (38.6)	5 (23.8)	10 (55.6)	
Bacteremia	5 (12.8)	2 (9.5)	3 (16.7)	
Cutaneous abscess	7 (17.9)	4 (19)	3 (16.7)	
Lymphadenopathy	5 (12.8)	4 (19)	1 (5.5)	
Pericardial effusion	7 (17.9)	6 (28.7)	1 (5.5)	
2. Pulmonary nocardiosis	176 (81.9)	76 (78.4)	100 (84.7)	
Pleural effusion	18 (10.2)	9 (11.8)	9 (9.0)	
**Antibiotic treatment**				
Combination therapy (≥ 2 drugs)	90 (41.9)	30 (30.9)	60 (50.9)	0.004
TMP-SMX plus imipenem TMP-SMX plus amikacin TMP-SMX plus ceftriaxone TMP-SMX/imipenem/amikacin Imipenem plus amikacin		15 (50.0) 6 (20.0) 2 (6.7) 5 (16.6) 2 (6.7)	47 (78.4) 4 (6.7) 5 (8.3) 2 (3.3) 2 (3.3)	
Monotherapy: TMP-SMX	98 (45.6)	55 (56.7)	43 (36.4)	
**Pattern of chest radiograph**				
Patchy infiltration Cavity Consolidation Interstitial infiltration Nodule	102 (47.4) 52 (24.2) 27 (12.5) 24 (11.2) 10 (4.7)	47 (48.5) 27 (27.8) 11 (11.3) 12 (12.4) 0	55 (46.6) 25 (21.2) 16 (13.6) 12(10.1) 10 (8.5)	

Fever and cough are the most common symptoms, with the median (IQR) duration of illness before admission being 30 (16) and 7 (11) days for patients with and without AIDS, respectively. Disseminated nocardiosis and pleural effusion were more prevalent among AIDS patients, whereas non-AIDS patients exhibited more severe complications, including shock and respiratory failure, and were more likely to develop brain abscesses. Multi-lobar infiltration was more common in patients without AIDS, whereas chest radiographs frequently revealed diffuse infiltration in both groups. Of the 150 patients who underwent brain imaging, 15 (10%) had brain abscesses; five had AIDS; and ten did not. Seizures were the predominant symptom. The majority of them (73.3%) received combination antimicrobial therapy; TMP-SMX and imipenem were the most frequent prescribing regimens (53.3%). Six (40%) of them die.

Trimethoprim-sulfamethoxazole (TMP-SMX) was the main antibiotic therapy for nocardiosis. Individuals who had a documented history of drug allergies were not prescribed TMP-SMX. Non-AIDS patients significantly more frequently received combination antibiotic therapy (50.9% vs. 30.9%, *p* value = 0.004), with TMP-SMX and imipenem being the most often prescribed regimens. AIDS patients commonly received TMP-SMX monotherapy (55, 56.7%) (Table 2). The median duration of treatment for nocardiosis was 12 and 6 months (range 6–12) for patients with and without AIDS, respectively. After 6 months of TMP-SMX monotherapy, two non-AIDS patients with pulmonary nocardiosis relapsed within a year. The common side effect of choosing TMP-SMX was that three patients developed severe skin rash and one developed pancytopenia.

**Fig. 2:**
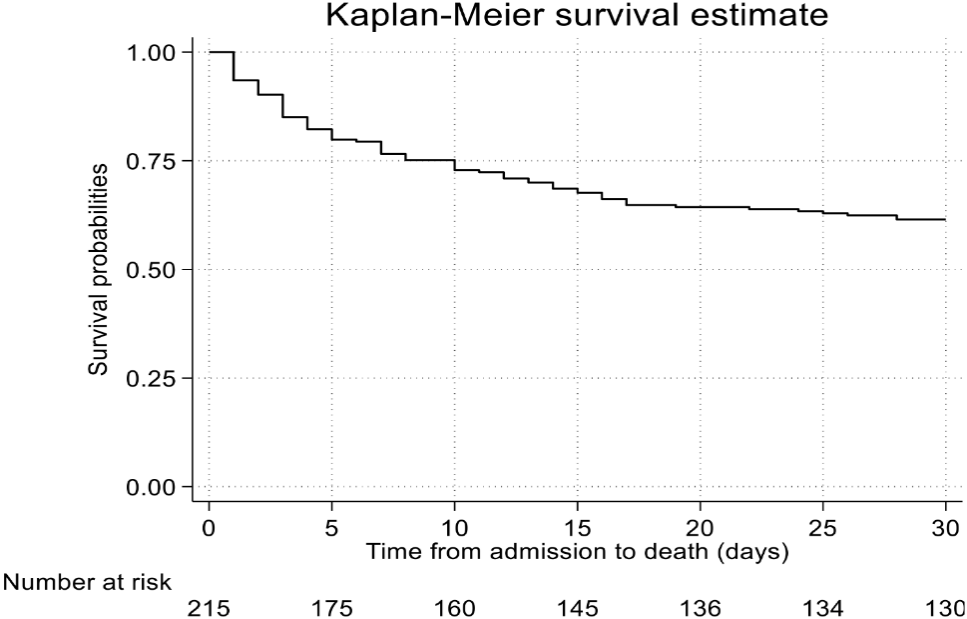
Survival plots for all pulmonary nocardiosis patients

**Fig. 3:**
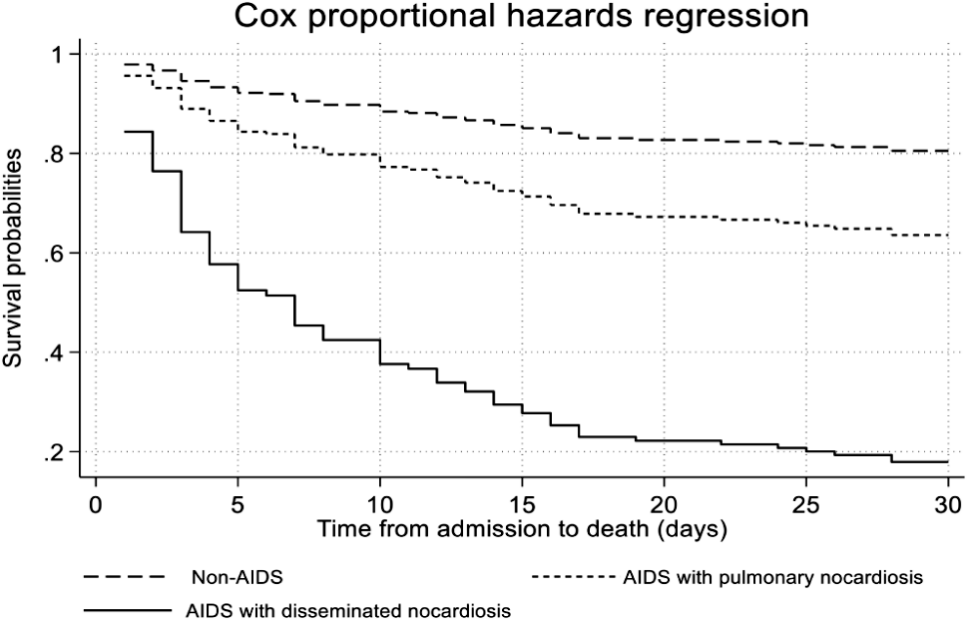
Adjusted survival curves based on the Cox proportional hazards model comparing AIDS and non-AIDS patients

The overall 30-day mortality rates in patients with pulmonary nocardiosis were 38.5% (95% CI: 32.3%, 45.4%). The Kaplan-Meier survival curve for 30-day mortality is illustrated in Fig. 2. The crude 30-day mortality rate was greater among non-AIDS patients (44.9%) than among AIDS patients (36.1%). When other factors like age, Charlson comorbidity index, concurrent opportunistic infections, length of illness, shock, respiratory failure, multi-lobar pneumonia, lung abscesses, and combination antibiotic therapy were taken into account, the risk of death within 30 days was 7.93 times (95% CI, 2.61–24.02, *p* < 0.001) for AIDS patients with disseminated nocardiosis and 2.09 times (95% CI, 0.74–5.87, *p* = 0.162) for AIDS patients with pulmonary nocardiosis compared to non-AIDS patients (Table 3; Fig. 3). Additionally, the overall 1-year mortality rate was 52.1% (95% CI: 45.6%, 58.9%).

**Table 3:** Cox proportional hazard analysis of patients with pulmonary nocardiosis

Factor	Person-time	Mortality rate/ 100 person	Crude HR (95% CI)	Adjusted HR^1^ (95% CI)	*P* value
Non-AIDS (*N* = 118)	2485	1.97	1	Ref.	
AIDS with pulmonary nocardiosis (*N* = 76)	1809	1.16	0.62	2.09 (0.74, 5.87)	0.162
AIDS with disseminated nocardiosis (*N* = 21)	270	4.44	1.97	7.93 (2.61, 24.02)	< 0.001

HR: Hazard ratio

1.adjusted for age, charlson comorbidity index, concurrent opportunistic infection, duration of illness, shock, respiratory failure, multi-lobar pneumonia, lung abscess, and combination antibiotic therapy

## Discussion

*Nocardia* species infection is an opportunistic infection in immunocompromised patients. AIDS is the most prevalent compromised underlying condition in the present study (45%), which is higher than other studies [7, 8]. Most of them had a CD4 count less than 100 cells/mm^3^, the same as in a previous study [9]. One-fifth of nocardiosis patients in an Australian study had no obvious predisposing condition [10], while in this study it was 4.2%.

In most studies, pulmonary nocardiosis is the most prevalent manifestation of infection due to *Nocardia* species [8, 11], including our study (224/237, 94.5%). To the best of our knowledge, this study is the first to compare the clinical outcome of pulmonary nocardiosis between AIDS and non-AIDS patients. Pulmonary nocardiosis should generally be considered in the differential diagnosis of subacute to chronic pneumonia. However, the duration of illness in non-AIDS patients in the present study was significantly shorter than in AIDS patients, mostly less than 14 days. Patchy alveolar infiltration and lung cavities were frequent chest radiographic findings in our study and a prior study conducted in Thailand [12].

Disseminated nocardiosis in this study was 18.1%, which is lower than the previous study [1, 13]. Brain abscess was the most common extrapulmonary nocardiosis in a study from 1994 (44%) [1]; therefore, central nervous system (CNS) involvement should always be suspected in immunocompromised patients, even if there are no neurological symptoms. In this study, the prevalence of brain abscess in patients with pulmonary nocardiosis was much lower (10%). Nevertheless, only 150 out of 215 patients had brain imaging. Further research is needed to establish the most appropriate approach for CNS evaluation in immunocompromised patients with nocardiosis.

The standard antimicrobial treatment regimen for nocardiosis remains absence of consensus regarding the optimal empirical treatment, depending on the *Nocardia* species and susceptibility pattern, which recommend testing by the broth microdilution method [14]. Worldwide, TMP-SMX is the main antibiotic therapy [2, 8], including in our study (184/215, 85.6%). This study’s limitations do not include investigating antimicrobial susceptibility or identifying *Nocardia* species. However, based on the available data in Thailand, *N. asteroid*es is the most prevalent species within the respiratory system, which is typically sensitive to TMP-SMX [15]. In cases of disseminated disease, CNS involvement, or more severe diseases, Margalit I. et al. propose a multidrug regimen to increase the likelihood of having an active agent [16]. In the present study, 90 (41.9%) patients received a combination regimen, particularly non-AIDS patients who had a higher incidence of brain abscesses and more severe complications, as well as AIDS patients with disseminated disease.

The overall 30-day mortality rates observed in this study (38.5%) were greater than those of prior research [7, 9, 11], comparable to the mortality rate of nocardia bacteremia (40%) [9]. The crude mortality rate in AIDS patients was lower than in non-AIDS patients. Age seems to be the most crucial covariable. In the Cox survival analysis, people with AIDS and disseminated nocardiosis had a 7.93-fold (95% CI, 2.61–24.02, *p* < 0.001) higher risk of dying within 30 days than people without AIDS. The study took into account factors like age, Charleson comorbidity index, concurrent opportunistic infections, length of illness, shock, respiratory failure, multi-lobar pneumonia, lung abscesses, and combination antibiotic therapy. While AIDS and pulmonary nocardiosis had a tendency to die within 30 days, the statistical analysis was not significant (2.09 (95% CI, 0.74–5.87, *p* = 0.162)). The all-cause 1-year mortality rate in this study (52.1%) was higher than the previous studies (19%, 29.4%) [5, 8]. According to our findings, AIDS with disseminated disease has a high mortality rate; therefore, we suggest early diagnosis and at least two antimicrobial treatment regimens, even though data on antimicrobial susceptibility and nocardia species are not available.

## Conclusions

AIDS-associated pulmonary nocardiosis, particularly disseminated nocardiosis, had a higher mortality rate compared to non-AIDS patients when adjusted for age and other risk factors. In resource-limited settings, early diagnosis and a combination of antimicrobial therapy may be the appropriate management.

## Data Availability

Not applicable.
